# The Diseases of Children

**Published:** 1881-06

**Authors:** 


The Diseases of Children. A practical and systematic work for practitioners and students. By William Henry Day, M.D., Physician to the Sanitarian Hospital for Women and Children, etc., etc. Second edition. Revised and much enlarged. Presley Blakiston, Philadelphia. 1881.

This is a complete treatise on diseases of children, comprising over seven hundred pages of subject matter. Diseases with their varieties are classed after the method sustained by modern pathologists, and the treatment, based on clinical experience and observation, is discussed on this basis to the exclusion of theoretical obscurities.

Hygienic principles are given their proper prominence in the management of children, and the constitutional state is considered in the indications of disease in preference to trusting to certain physical and local signs.

